# The Role of Citrullination Modification in CD4^+^ T Cells in the Pathogenesis of Immune-Related Diseases

**DOI:** 10.3390/biom14040400

**Published:** 2024-03-26

**Authors:** Yuhang Chen, Yi Teng, Ping Xu, Shengjun Wang

**Affiliations:** 1 Department of Laboratory Medicine, Affiliated Hospital of Jiangsu University, Zhenjiang 212001, China; sebastianchen1013@163.com; 2 Jiangsu Key Laboratory of Laboratory Medicine, Department of Immunology, School of Medicine, Jiangsu University, Zhenjiang 212013, China; 3 Department of Laboratory Medicine, The Fifth People’s Hospital of Suzhou, Suzhou 215505, China

**Keywords:** citrullination, T cells, immune disorder, disease

## Abstract

The post-translational modifications (PTMs) of proteins play a crucial role in increasing the functional diversity of proteins and are associated with the pathogenesis of various diseases. This review focuses on a less explored PTM called citrullination, which involves the conversion of arginine to citrulline. This process is catalyzed by peptidyl arginine deiminases (PADs). Different members of the PAD family have distinct tissue distribution patterns and functions. Citrullination is a post-translational modification of native proteins that can alter their structure and convert them into autoantigens; thus, it mediates the occurrence of autoimmune diseases. CD4^+^ T cells, including Th1, Th2, and Th17 cells, are important immune cells involved in mediating autoimmune diseases, allergic reactions, and tumor immunity. PADs can induce citrullination in CD4^+^ T cells, suggesting a role for citrullination in CD4^+^ T cell subset differentiation and function. Understanding the role of citrullination in CD4^+^ T cells may provide insights into immune-related diseases and inflammatory processes.

## 1. Introduction

**Figure 1:**
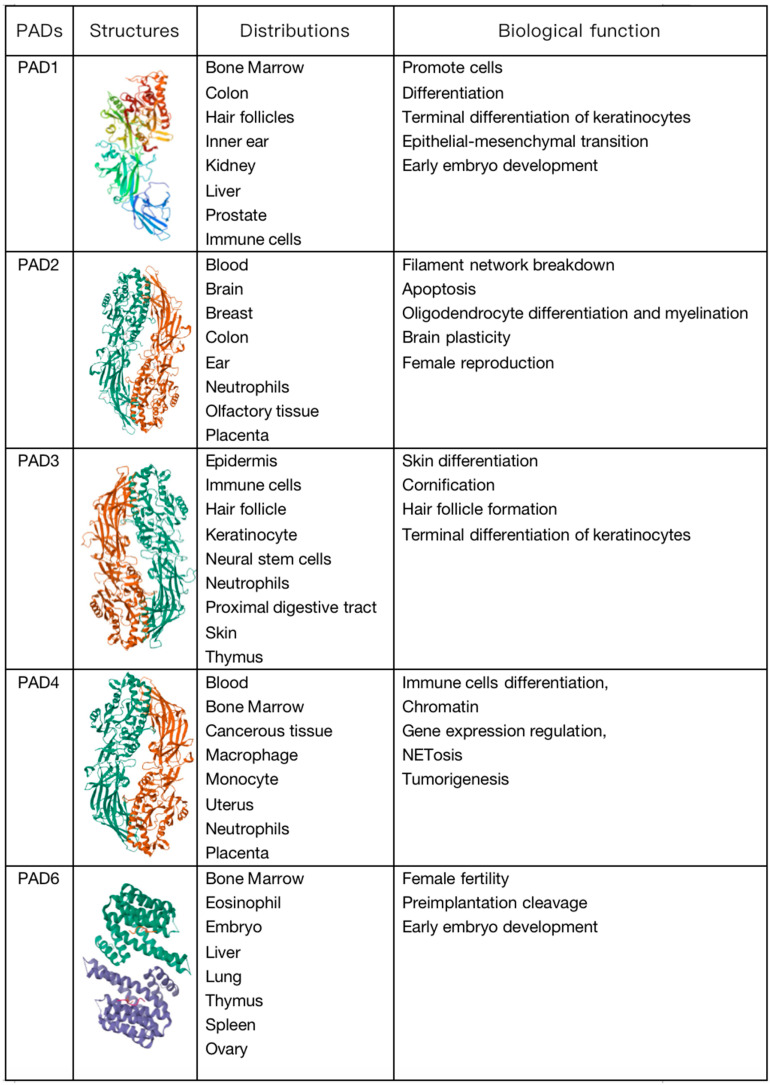
Molecular structure, distribution, and biological functions of peptidyl arginine deiminase (PAD) enzymes.

**Figure 2:**
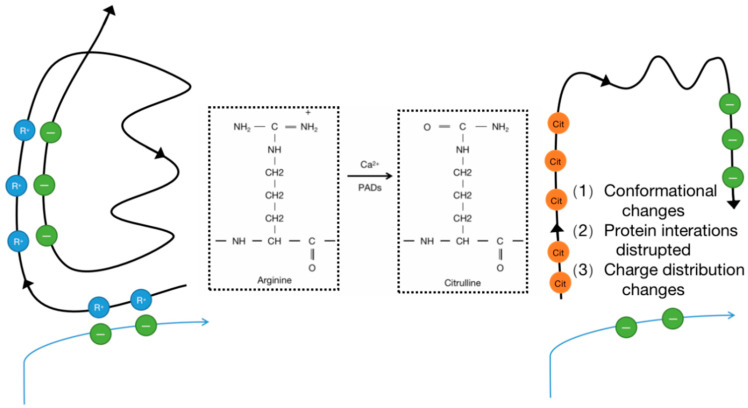
Enzymatic conversion of positively charged arginine into neutral citrulline and the PTM may also promote new protein interactions.

The expression of proteins is regulated by several layers of overlapping control mechanisms. Transcriptional, post-transcriptional, and translational control mechanisms determine whether a protein is expressed in a specific cell at a specific time. Once translated, proteins can undergo chemical modifications through enzymatic and non-enzymatic reactions. These post-translational modifications (PTMs) can influence the structure, stability, subcellular localization, and activity of proteins, and they can also regulate their affinity for binding to other proteins, metabolites, and nucleic acids. Protein modifications can be controlled spatially and temporally, enabling cells to respond to changes in their environment such as stress signals, developmental cues, variations in nutrient or oxygen availability, and carcinogenic damage. Thus, PTMs expand the functional proteome far beyond the complexity of the genome and add a significant level of complexity to biological systems, endowing them with responsiveness and adaptability [1]. In other words, PTMs can be considered a means to control the collective metabolism of cells by altering the structure and function of proteins. To date, over 200 types of PTMs have been described; the most common PTMs include phosphorylation, methylation, acetylation, ubiquitination, and glycosylation [2]. Citrullination is a protein PTM that has historically been less explored but has gained increasing research attention. It is an irreversible PTM since there is currently no evidence indicating the existence of a de-citrullination reaction. In this reaction, the positively charged guanidino group of arginine is replaced by a ketone group, resulting in a neutrally charged citrulline residue. Therefore, this process is also referred to as a deamination reaction. This process is catalyzed by peptidyl arginine deiminases (PADs), which constitute a calcium-dependent and highly conserved enzyme family that includes PAD1–4 and PAD6 (Figure 1) [3]. PADs were first reported in 1977 as citrullination enzymes during hair growth with their substrate being hair keratin [4]. Mammalian PADs isoenzymes share 70~95% homology in amino acid sequences, consisting of approximately 663 amino acids, with a molecular weight of approximately 74 kDa [5]. Each member of the family has a unique pattern of organizational distribution and function, including cellular differentiation, neural growth, apoptosis, inflammation, gene regulation, and early embryonic development. Each PAD has a distinct tissue distribution pattern. For example, hematopoietic cells predominantly express PAD2 and PAD4 with the highest expression level of PAD4 in neutrophils. PAD6 is almost exclusively expressed in oocytes, while PAD1 and PAD3 are primarily expressed in nonhematopoietic cells [6]. Evidence suggests that PADs can influence tumor development by regulating cell signaling, transcription, and the extracellular matrix (ECM), thereby modulating growth, apoptosis, and epithelial–mesenchymal transition (EMT) [7]. Citrullination is a hydrolytic reaction that increases the mass of a protein by 0.98 Da while decreasing its positive charge. Citrullination significantly reduces the amino acid’s isoelectric point. The isoelectric point for arginine is 11.41, whereas for citrulline, it is 5.91. This alteration affects the protein’s acidity and its potential to form hydrogen bonds and electrostatic interactions between amino acids [8]. In other words, a citrullination-induced loss of positive charge leads to electrostatic and conformational changes in modified proteins, altering the distribution of protein charges, hindering hydrogen bond formation, and increasing protein hydrophobicity, profoundly affecting protein structure and folding (Figure 2). This alteration affects protein function by modifying binding sites, protein–protein interactions, and degradation sensitivity [6,8,9]. In fact, like other PTMs, citrullination has been shown to impact several aspects of protein biology, such as the structure, stability, localization, protein–nucleic acid binding, and catalytic activity, and it can also influence the subsequent deposition of other PTMs. Since citrulline is a non-coded amino acid, its presence in proteins can only occur through modification, implying cellular state or environmental changes and the initiation of relevant responses [7]. Various proteins, including cytoplasmic, nuclear, membrane, and mitochondrial proteins, can undergo citrullination [10]. The human “citrullinome” comprises hundreds of proteins with the most prominent being structural proteins, including keratins, fibronectin, actin, tubulin, and myosin; and chromatin proteins, particularly histones [11]. Recently, citrullinated proteins have garnered significant scientific attention due to their involvement in several crucial biological processes, including skin keratinization, myelin formation, and gene expression regulation [12,13]. Citrullinated proteins can be detected by antibody-based detection systems. Although citrullination can occur relatively easily, not all arginine residues in proteins can be citrullinated. For example, arginine residues in β-turns are more prone to citrullination than are those in α-helices, and arginine positioned adjacent to proline or glutamine significantly reduces the likelihood of citrullination [14]. Notably, free arginine cannot be citrullinated by PADs [3,15]. PADs are considered to be involved in the development of various autoimmune diseases such as rheumatoid arthritis (RA), lupus, ulcerative colitis (UC), Alzheimer’s disease, multiple sclerosis (MS), etc. The abundant abnormal citrullination of self-proteins has been detected in the affected tissues and peripheral blood of patients with these diseases [16]. Some naturally occurring proteins, when translated through citrullination modification sites, may increase susceptibility to loss of tolerance, thereby transforming into autoantigens capable of evading immune tolerance [17], and multiple studies have shown that an excess of citrullinated proteins is a characteristic of most immune disorders [16]. Research suggests that citrullination, by altering the structure and protein hydrolysis of citrullinated antigens, can globally alter antigen processing and presentation, facilitating the generation and presentation of novel cryptic epitopes capable of stimulating autoimmune reactive T cells in rheumatoid arthritis (RA) patients [18].

The development process of T cells in the thymus has been extensively studied. Hematopoietic stem cells in the bone marrow differentiate into lymphoid progenitor cells, which, upon entering the thymus through blood vessels at the corticomedullary junction, retain the potential to differentiate into other lineages. Subsequently, the expression levels of Notch receptors increase, initiating the differentiation of cells toward the T cell lineage. Based on the surface expression of CD4 and CD8, T cell development in the thymus progresses through three stages: Double Negative (DN), Double Positive (DP), and Single Positive (SP) cells. Upon maturation, T cells exit the thymus and migrate to peripheral immune tissues such as the spleen and lymph nodes. T cells that have not encountered antigens remain in a quiescent state and are referred to as naive T cells. The activation and differentiation of effector T cells and memory T cells occur through interactions with antigen-presenting cells. Based on their functions, effector T cells can be further categorized into CD4^+^ helper T cells (including Tfh cells), CD8^+^ cytotoxic T lymphocytes (CTLs), and regulatory T cells (Tregs). Cytotoxic T cells (CTLs), also known as CD8^+^ cytotoxic T lymphocytes, are the primary effector cells involved in antitumor immunity.

CD4^+^ T cells are important immune cells in the human immune system. They play a crucial role in mediating adaptive immunity against various pathogens. The function of CD4^+^ T cells depends on the T cell receptor (TCR), the activation of which triggers the release of Ca^2+^ from the endoplasmic reticulum. Ca^2+^ acts as a second messenger in cells, participating in multiple biological processes such as cell cycle progression and proliferation by altering its concentration inside and outside the cell to maintain the normal function of immune cells [19]. They are also involved in autoimmune diseases, asthma, allergic reactions, and tumor immunity. CD4^+^ T cells not only play a crucial supporting role in the activation of CTLs but also actively produce cytokines and chemokines, indirectly contributing to autoimmune diseases, asthma, allergic reactions, and tumor immunity [20]. CD4^+^ T cells are important mediators of immune memory, and when their numbers or functions decrease, individuals become more susceptible to various infectious diseases [20]. CD4^+^ T cells can be activated by different stimuli and differentiate into corresponding T cell subsets. Based on the differences in cytokine production, activated CD4^+^ T cells are mainly divided into Th1, Th2, Th17, T follicular helper cells (Tfh), and regulatory T cells (Tregs). Th1 cells enhance macrophage-mediated immune responses to intracellular pathogens, mediating cell-mediated immune responses. Th2 cells stimulate the activation of mast cells, eosinophils, and basophils, exerting humoral immune functions, and mediating immune responses to parasites. Th17 cells primarily target extracellular bacterial immune responses and can stimulate the recruitment of neutrophils to the site of infection. Tfh cells stimulate B cell maturation in germinal centers. Treg cells inhibit immune responses to maintain immune homeostasis [20,21,22]. Previous studies have reported that guanine nucleotide peptides can stimulate the proliferation of existing CD4^+^ T cells and induce oligo-clonal TCR expansion, indicating that stress, inflammation, and/or infection can induce protein citrullination on CD4^+^ T cells [23].

All in all, while citrullination modification can to some extent mediate autoimmune diseases by affecting both native proteins and T helper cells, there are still distinctions that need to be made between the two. This review aims to explore the impact of citrullination modification on CD4^+^ T cells themselves, including their differentiation, function, and involvement or mediation in autoimmune diseases.

## 2. Role of Citrullination Modification in CD4^+^ T-Cell Subset Differentiation

**Figure 3:**
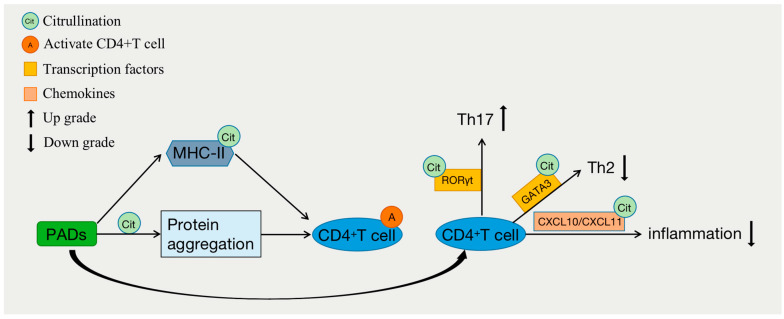
Citrullination and differentiation of CD4^+^ T cells.

The development and differentiation of CD4^+^ T cells are closely associated with the occurrence and progression of various immune-related diseases, such as cancer, autoimmune diseases, and hypersensitivity reactions. Under the influence of internal and external factors, CD4^+^ T cells can further differentiate into distinct subsets, thereby exerting diverse immune functions [24]. For example, they can differentiate into Th1 cells, which are involved in combating intracellular viral or bacterial infections and antitumor immunity; Th2 cells, which participate in allergic reactions and defense against parasitic infections; or Th17 cells, which play a role in fighting extracellular bacterial and fungal infections and contribute to the pathogenesis of various inflammatory diseases when excessively or persistently activated. CD4^+^ Th cells play a critical role in immune defense against pathogen invasion, but if their functions are not properly regulated, they can lead to autoimmune and allergic diseases [25]. Studies have shown that epigenetic regulation plays an important role in the development and differentiation of T cells (Figure 3) [26]. The differentiation of CD4^+^ T cells relies on the regulation of several lineage-specific transcription factors, such as T-bet for Th1 cells, GATA3 for Th2 cells, and RORγt for Th17 cells. The post-translational citrullination of transcription factors has been reported to regulate the differentiation of CD4^+^ T cells. PAD2 can induce the citrullination of GATA3 and RORγt, severely impacting the differentiation of Th2 and Th17 cells. Citrullination of the R330 site in GATA3 suppresses its ability to bind DNA, while citrullination of the four arginine residues in RORγt enhances its ability to bind DNA. The inhibition of citrullination can enhance Th2 differentiation but weaken Th17 cell differentiation, thereby increasing susceptibility to allergic airway inflammation [6]. In contrast, the overexpression of PAD2 in human peripheral blood mononuclear cells reduces the polarization of Th2 cells and increases the polarization of Th17 cells [27]. Similarly, research has indicated that PAD2-mediated citrullination can promote the activation of Th17 cells and the production of cytokines such as IL-6, IL-17A, IL-17F, IL-21, and IL-22 through endoplasmic reticulum stress and autophagy while inhibiting the production of IL-4 and IL-13, thereby suppressing Th2 activation [28]. Interestingly, despite its lack of association with Th1 polarization, inhibiting PAD2 can attenuate IFN-γ production in Th1 cells [6]. In addition to directly altering the function and polarization of CD4^+^ T cells, PAD2 can also influence the activity of T cells by citrullinating certain chemokines involved in T-cell chemotaxis, such as CXCL10 and CXCL11 [29]. T cells exhibit decreased sensitivity to citrullinated CXCL10 and CXCL11, resulting in a reduced migration of T cells to inflammatory sites and a subsequent diminished inflammatory response. Recent studies have indicated that the differentiation of helper T cells, a process that could be mediated by citrullination, is also influenced by network-associated extracellular proteins [30].

## 3. Role of Citrullination Modification in the Regulation of CD4^+^ T-Cell Function

CD4^+^ T lymphocytes are important immune cells in the human immune system, and their function depends on the activation of T-cell receptors (TCRs). The activation of TCRs triggers the release of Ca^2+^ from the endoplasmic reticulum. Ca^2+^ acts as a second messenger in cells and is involved in various biological processes, such as cell cycle progression and proliferation, to maintain the normal function of immune cells [19]. The PADs that mediate citrullination require the presence of Ca^2+^, suggesting that citrullination may regulate the function of CD4^+^ T cells (Figure 3). Major histocompatibility complex (MHC) class II antigen processing is a complex process that is strongly affected by protein structures in the entry pathway [31]. Studies have shown that even minor changes in proteins, such as those introduced by post-translational modifications (PTMs), can have profound effects on protein structure and alter the susceptibility of residues to key proteases. Overall, these changes can greatly impact downstream antigen processing and result in the generation of unique T-cell epitopes [32,33,34]. Citrullination occurs during cellular stress and/or apoptosis and can result in protein aggregation and the stimulation of CD4^+^ T cells and antigen responses. Protein degradation and recycling can also occur during autophagy, which is a process that induces MHC-II expression in stressed cells and inflammatory processes. Citrullination epitopes on MHC-II can stimulate CD4^+^ T-cell responses [35]. Research has also shown that citrullinated peptide epitopes can be presented on MHC-II through autophagy and PAD-dependent mechanisms in antigen-presenting cells (APCs) and epithelial cells [36]. Earlier studies have shown that citrullinated modifications occurring at the peptide side chain positions of vimentin and the human protein polysaccharide aggrecan interact with shared epitopes, significantly increasing peptide–MHC binding affinity; in one experiment, these modifications led to the activation of CD4^+^ T cells in DR4 transgenic mice [37].

## 4. Role of Citrullination Modification in Immunomodulation, Immune Tolerance, and Autoimmune Diseases

Autoimmune diseases encompass a wide range of conditions in which the immune system targets and destroys host-derived proteins (self-antigens) [38]. The determining factors for processing and effective presentation within the groove of MHC class II are referred to as “dominant”, while those factors that remain unexposed (most determinants) are termed “cryptic”. For self-antigens, only dominant epitopes emerge during the thymic T-cell tolerance development process, thereby preserving a pool of CD4^+^ T cells recognizing the cryptic epitopes [39]. As, in most instances, the output of antigen processing and presentation for a specific antigen within a particular individual remains constant, the recognition of the cryptic epitopes by T cells rarely encounters its specific self-antigen. However, alterations in the processes or environment generating dominant and cryptic epitopes may allow determinants to emerge, activating CD4^+^ T cells. For instance, modifications during early protein hydrolysis events in the antigen-processing pathway could disrupt dominant epitopes and/or allow the appearance of previously ineffective cryptic epitopes, rendering them non-tolerant. Furthermore, modifications or mutations of specific amino acids could generate new dominant epitopes, initiating an effective T cell response against self-antigens [40]. Post-translational modifications of self-antigens may play a role in autoimmunity by inducing conformational changes that potentially alter the processing of self-antigens, thereby initiating and driving autoimmune responses [41]. However, as mentioned earlier, citrullination robustly affects protein structure and folding [6,8,9], and research has confirmed that citrullination can regulate the processing of MHC-II antigens through the generation and destruction of epitopes, resulting in the loss of immune tolerance to citrullinated proteins. Moreover, most changes in antigen processing caused by citrullination lead to the generation of new or enhanced original antigenic epitopes, possibly leading to extensive alterations in the self-antigenic peptide repertoire produced by antigen-presenting cells (APCs) [18]. Citrullination is prevalent in autoimmune diseases and cancer, such as rheumatoid arthritis (RA) [42,43], diabetes [44,45], psoriasis [46], Alzheimer’s disease (AD) [47,48,49], multiple sclerosis [12,50] and various cancers [13]. The research results showed that the culture of PBMCs from patients with citrullinated MBP (Myelin basic protein) 87–99 APL induces strong and uniform Th1 polarization, indicating the crucial role of citrullination in the antigen recognition of MS T cells at self-epitopes [51]. Currently, we are focusing on the role of CD4^+^ T cells in citrullination-induced autoimmune diseases with an emphasis on rheumatoid arthritis (RA). Rheumatoid arthritis is an autoimmune chronic inflammatory disease that primarily affects the synovium of multiple joints, leading to inflammation, proliferation, and eventual destruction. Without timely treatment, it can progress to the destruction of joint cartilage, resulting in deformity and functional disability, significantly reducing the quality of life for patients. Although the main lesions occur in the synovium of peripheral joints, in at least 40% of rheumatoid arthritis patients, this disease may affect other organs, manifesting as specific or nonspecific extra-articular inflammatory presentations, with systemic implications [52]. Citrullination is considered a major cause of the onset of RA, and citrullinated proteins are the primary targets of antibodies in RA patients [53]. The pathogenesis of rheumatoid arthritis (RA) is associated with an increase in citrullinated protein levels mediated by PAD2 in synovial fluid [54]. B cells can recognize citrullinated epitopes and produce autoantibodies against citrullinated proteins [55,56]. Increased levels of anti-citrullinated protein antibodies (ACPAs) can be detected in 70% of RA patients. Following treatment with anti-rheumatic drugs, the levels of circulating ACPAs are lowered and are correlated with a decrease in the severity of RA [57,58]. These results suggest that citrullinated proteins regulated by PAD2 contribute to enhanced inflammatory responses in RA patients. Citrullination can affect the recognition of APCs, T cells, and B cells by enabling the formation of new epitopes and increasing the exposure of citrullinated antigens, thereby facilitating the uptake and recognition of citrullinated antigens by APCs. In RA patients harboring the HLA-DRB1 shared epitope (SE), both T cells and B cells can recognize citrullinated antigens [59]. B cells in RA can act as antigen-presenting cells (APCs) and recognize citrullinated antigens through binding to specific receptors on B-cell surfaces, promoting the differentiation of T cells into memory T cells. Activated T cells can then stimulate the release of anti-citrullinated protein antibodies (ACPAs) from B cells [60]. A previous study has revealed the previously unknown clonal expansion of cytotoxic CD8^+^ T cells in the blood and synovium of ACPA^+^ RA patients. These cytotoxic CD8^+^ T cells exhibit proinflammatory and cytolytic mediator expression. The authors demonstrated that guanine-modified antigens activate these cytotoxic CD8^+^ T cells in an HLA class I-dependent manner, leading to their expansion, effector activity, and the killing of target cells [61]. Additionally, memory T cells with tissue-resident memory (TRM) features, characterized as SF CD69^+^CD103^+/−^CD8^+^ cells, play a role in the pathogenesis of ACPA-positive RA by inducing the formation of citrullination proteins [62]. Healthy donors have previously been shown to have citrulline-specific CD4^+^ T cells and CD4-specific responses [63]. In the synovium of RA patients, there is a significant infiltration of CD4^+^ T cells, and studies have shown that citrulline-specific T cells isolated from RA patients mainly exhibit Th1 and Th17 phenotypes. Th1 cells activate macrophages to act as antigen-presenting cells (APCs) and release proinflammatory cytokines, such as IFN-γ, interleukin-2 (IL-2), and TNF-α. Th17 cells can stimulate RA synovial fibroblasts to produce proinflammatory cytokines, chemokines, and matrix metalloproteinases. They also promote angiogenesis in the synovial membrane and osteoclast differentiation through the secretion of IL-17 [64]. Disrupted peptidyl arginine deiminase (PAD) activity and the aberrant expression of citrullinated proteins, including citrullinated filaggrin, histones, and transcription factors, have been observed in the synovial fluid of rheumatoid arthritis patients [42,43]. Evidence suggests that citrullinated GRP78 is an autoantigen associated with rheumatoid arthritis and type 1 diabetes, and CD4^+^ T cells that are reactive to GRP78 have been identified [65]. Under steady-state conditions, the outcome of this physiological citrullination modification may be peripheral tolerance. However, under conditions of inflammation and endoplasmic reticulum stress, peripheral tolerance may be disrupted, and the expression of GRP78 may increase [66]. The development of rheumatoid arthritis also involves the presence of anti-citrullinated protein antibodies (ACPAs). Citrullinated proteins and ACPAs can induce arthritis. Citrullinated fibrinogen can promote the proliferation of peripheral blood mononuclear cells (PBMCs) in RA, while noncitrullinated fibrinogen does not exert a proliferative effect on RA PBMCs. Both citrullinated proteins and the HLA-DRB1 shared epitope (SE) are essential factors in the pathogenesis of arthritis. In RA, ACPAs activate through both the classical and alternative pathways, working in conjunction to recruit immune cells, increase vascular permeability, promote phagocytosis, and release proinflammatory cytokines and chemokines. This process leads to tissue damage and further promotes citrullination [67]. The citrullination of MBP enhances the presentation of MBP85–99 and induces the generation of TNF-α-producing Th cells in HLA-DR15^+^ MS patients. Additionally, hypercitrullinated MBP can also induce the differentiation of CD4^+^ T cells into Th17 cells, suggesting that citrullination may play a role in disrupting tolerance to this autoantigen [68]. The occurrence of autoimmune diseases is closely tied to the loss of immune tolerance, and by altering the structure and protein hydrolysis of citrullinated antigens, citrullination is believed to comprehensively modify antigen processing and presentation, promoting the generation and presentation of new cryptic epitopes capable of stimulating autoreactive T cells in RA patients [18]. Experimental evidence suggests that citrullination contributes to the breakdown of immune tolerance and may generate neoantigens, which can become additional targets during epitope spreading [69]. The loss of immune tolerance mediated by citrullination can also occur in periodontal disease (PD). PD is an oral inflammatory disease that affects the tissues supporting the teeth and, if left untreated, can lead to tooth loss. PD is caused by Gram-negative anaerobic bacteria, specifically *Porphyromonas gingivalis* [12,70]. *Porphyromonas gingivalis* peptidyl arginine deiminase (PPAD) rapidly citrullinates bacterial and host proteins, such as fibrinogen and α-enolase [12]. Released PPAD can disseminate into the host’s connective tissues, where it citrullinates and modifies epidermal growth factor (EGF), preventing its recognition by epithelial cells. This mechanism delays local healing processes and disrupts the protective epithelial barrier in periodontal tissues [70]. PPAD also generates a set of novel highly antigenic epitopes. When an excessive amount of these epitopes is present, tolerance barriers can be breached, leading to the production of anti-citrullinated protein antibodies (ACPAs) and potentially triggering acute autoimmune responses [70,71]. Experimental and clinical research has clearly indicated that increased citrullination in PD patients may be mediated by both PPADs and host PADs. This could lead to the breakdown of immune tolerance and subsequent increase in the release of autoantibodies characteristic of rheumatoid arthritis (RA), which genetically predispose individuals to RA, thus triggering the onset of RA [72,73,74,75]. High levels of ACPAs can be observed in patients with aggressive periodontitis [76]. Although periodontal disease itself is not an autoimmune disease, it can induce severe autoimmune responses in the host, with citrullinated proteins being an important factor in disease progression. However, the researchers examined the reactivity of infiltrating CD4^+^ T cells in the cerebrospinal fluid (CSF) to citrullinated and unmodified MBP peptides. They found that these T cells showed minimal reactivity to citrullinated peptides, while their response to the unmodified version was slightly stronger, suggesting that citrullination may not be a significant activating factor for T cell responses in MS. Based on the experimental data, the researchers hypothesized that citrullination may be a consequence of immune or inflammatory responses rather than a primary driver of disease activation [77]. Our proposed explanation is that perhaps the unique distribution of citrullinated proteins in the brain tissue of MS patients, as well as the differences between CSF and other body fluids, contribute to the potential association between citrullination and the immunopathological processes of MS. However, it may not be the primary driving force behind T cell responses. Further research is needed to investigate the underlying reasons for this phenomenon.

## 5. Application of Citrullination Modification in Disease Treatment

The process of citrullination is associated with many human diseases and inflammation, leading to autoimmune responses against citrullinated proteins. In recent years, there has been substantial progress in understanding the role of citrullination in immune dysregulation. Increasing evidence emphasizes the importance of citrullination and the recognition of autoantibodies against citrullinated peptides or proteins, greatly expanding our understanding of the pathogenesis and clinical features of various immune-related diseases [16]. Several studies have demonstrated that modifying citrullination can mitigate inflammation under various inflammatory conditions [78,79,80,81] and may have applications in cancer therapy [82]. There are apparent approaches to interfere with citrullination and its various roles in inflammation, one such method involving the use of PAD (peptidyl arginine deiminase) inhibitors [83,84,85,86,87]. Many PAD inhibitors are used for the treatment of PAD-related diseases affecting the skin, joints, colon, and immune system [46,49,79,88]. The use of therapies that target citrullination is also increasing. Initially, Cl-amidine was the first PAD inhibitor tested in RA animal models [78]. These studies utilized collagen-induced arthritis models, and the data demonstrated that PAD inhibition could prevent the onset and occurrence of the disease [79]. The subsequent development of inhibitors with enhanced cellular potency and in vivo stability led to the synthesis of a series of compounds containing tetrazole and benzimidazole, including bb-Cl-amidine [89]. It is noteworthy that BB-Cl-amidine has demonstrated superior efficacy in lupus, RA, and type I diabetes models [89,90,91,92]. Nowadays, PAD inhibition has been proposed as a therapeutic strategy for autoimmune diseases, neurodegenerative diseases, and cancer because these disease states are often associated with abnormally high levels of citrullination. Research has suggested that inhibiting PAD can prevent diseases by modulating immune pathways [7,92]. Protein hypercitrullination in mouse models of neurodegenerative and autoimmune diseases, such as multiple sclerosis (MS), is closely linked to disease severity and progression. Treatment with PAD inhibitors reduces PAD enzyme activity in the brains and spinal cords of these mice, leading to reduced levels of citrullinated proteins and remyelination in the central nervous system [93]. Inhibiting PAD enzymes responsible for citrullination is a rational approach that can disrupt the citrullination pathway of proteins, thus affecting their various roles in inflammation [94]. However, PAD inhibition may have serious adverse effects on skin physiology, central nervous system development, gene regulation, immune system function, and the female reproductive system. Delivering PAD inhibitors to specific cells or tissues can reduce the risk of side effects and toxicity. For example, inhibiting PAD activity specifically within tumor cells through the internalization of tumor cell-targeting antibodies may be a means of inducing cell-specific cytotoxicity in these tumor cells. It is known that Pan-PAD inhibitors can alter the release of neutrophil extracellular traps (NETs) [95,96,97]. Research has indicated that BB-Cl-amidine reduces the spontaneous release of NETs from bone marrow-derived neutrophils in NOD mice, and these findings are consistent with studies on lupus and collagen-induced arthritis, suggesting that PAD inhibition reduces NET release and is associated with disease improvement [92]. Therefore, while the presence of citrullination inhibitors intervenes positively in the treatment of diseases, consideration should also be given to the adverse effects they may exert on the body. Future research efforts may perhaps focus on mitigating the extent of non-therapeutic side effects.

## 6. Conclusions

Citrullination is a widespread and essential biological phenomenon mediated by PADs that is involved in host immunity [98]. The process of citrullination is calcium dependent and is facilitated by PADs. Different PADs exhibit tissue-specific and substrate-specific characteristics, enabling them to catalyze citrullination in various tissues and substrates. Under physiological conditions, PADs remain inactive until they are stimulated by calcium. Once activated, these enzymes can citrullinate numerous structural proteins [99]. The conversion of arginine to citrulline results in a change in the amino acid’s charge, resulting in a neutral charge. This directly inhibits hydrogen bond formation, alters charge distribution, increases protein hydrophobicity, and it also adversely affects protein stability [99]. Citrullination is also considered a potential therapeutic target, as the conversion of peptidylarginine to peptidylcitrulline plays an important role in promoting tumorigenesis and autoimmune responses [92].

Aberrant citrullination can generate novel citrulline epitopes, leading to autoimmunity and potentially inducing conformational defects, damaging tissue structures, and exacerbating inflammation. This article summarizes the role and impact of PAD-mediated citrullination on the regulation of CD4^+^ T cell differentiation and function as well as on immune regulation and tolerance. This study also provides an overview of the potential pathogenic mechanisms related to citrullination in autoimmune diseases, especially RA. Additionally, the article offers some insights into therapies targeting citrullination.
